# Adiponectin accumulation in the retinal vascular endothelium and its possible role in preventing early diabetic microvascular damage

**DOI:** 10.1038/s41598-022-08041-2

**Published:** 2022-03-09

**Authors:** Taka-aki Sakaue, Yuya Fujishima, Yoko Fukushima, Yuri Tsugawa-Shimizu, Shiro Fukuda, Shunbun Kita, Hitoshi Nishizawa, Barbara Ranscht, Kohji Nishida, Norikazu Maeda, Iichiro Shimomura

**Affiliations:** 1 Department of Metabolic Medicine, Graduate School of Medicine, Osaka University, 2-2-B5, Yamada-oka, Suita, Osaka 565-0871 Japan; 2 Department of Ophthalmology, Graduate School of Medicine, Osaka University, 2-2-B5, Yamada-oka, Suita, Osaka 565-0871 Japan; 3 Integrated Frontier Research for Medical Science Division, Institute for Open and Transdisciplinary Research Initiatives, Osaka University, 2-2, Yamada-oka, Suita, Osaka 565-0871 Japan; 4 Department of Adipose Management, Graduate School of Medicine, Osaka University, 2-2, Yamada-oka, Suita, Osaka Japan; 5 Sanford Burnham Prebys Medical Discovery Institute, NIH-Designated Cancer Center, Development, Aging and Regeneration Program, La Jolla, CA USA; 6 Department of Metabolism and Atherosclerosis, Graduate School of Medicine Osaka University, 2-2, Yamada-oka, Suita, Osaka 565-0871 Japan

**Keywords:** Molecular biology, Diabetes, Diabetes complications, Endocrinology, Endocrine system and metabolic diseases, Metabolic syndrome

## Abstract

Adiponectin (APN), a protein abundantly secreted from adipocytes, has been reported to possess beneficial effects on cardiovascular diseases in association with its accumulation on target organs and cells by binding to T-cadherin. However, little is known about the role of APN in the development of diabetic microvascular complications, such as diabetic retinopathy (DR). Here we investigated the impact of APN on the progression of early retinal vascular damage using a streptozotocin (STZ)-induced diabetic mouse model. Our immunofluorescence results clearly showed T-cadherin-dependent localization of APN in the vascular endothelium of retinal arterioles, which was progressively decreased during the course of diabetes. Such reduction of retinal APN accompanied the early features of DR, represented by increased vascular permeability, and was prevented by glucose-lowering therapy with dapagliflozin, a selective sodium-glucose co-transporter 2 inhibitor. In addition, APN deficiency resulted in severe vascular permeability under relatively short-term hyperglycemia, together with a significant increase in vascular cellular adhesion molecule-1 (VCAM-1) and a reduction in claudin-5 in the retinal endothelium. The present study demonstrated a possible protective role of APN against the development of DR.

## Introduction

Adiponectin (APN), a protein specifically secreted from adipocytes, has been shown to possess various protective roles against metabolic disorders, including insulin resistance^1^, organ fibrosis^2,3^, and atherosclerotic cardiovascular diseases^4,5^. APN circulates abundantly in human bloodstream (1–30 μg/mL), but its concentration decreases under pathological conditions, such as obesity, especially visceral fat accumulation^6^, and type 2 diabetes^7^. We and others previously reported that APN protein, but not gene expression, exists in the aortic endothelium, heart, skeletal muscle, proliferative smooth muscle cells, and renal pericytes through binding to T-cadherin, a unique glycosylphosphatidylinositol (GPI)-anchored cadherin^8–11^. Moreover, APN exhibited its protective effects on atherosclerosis^8^, cardiac hypertrophy^9^, muscle regeneration^10^, and renal tubular injury^11^ in a T-cadherin-dependent manner.

The number of patients who suffer from type 2 diabetes mellitus has been increasing worldwide^12^. Diabetic retinopathy (DR), one of the major diabetic microvascular complications, is a leading cause of vision loss in patients with diabetes^12^. Chronic hyperglycemia causes irreversible vascular abnormalities, followed by impaired neuronal function. Of note, early breakdown of the blood–retinal barrier (BRB), induced by endothelial injury and altered cell–cell interactions, has been postulated as the underlying mechanism of disease progression of DR^13^. Although there is evidence that APN deficiency resulted in the severe renal phenotype of diabetic nephropathy in streptozotocin (STZ)-induced diabetic model mice^14^, little is known about the physiological significance of APN in the development of DR.

Here, we demonstrate T-cadherin-dependent APN accumulation in the murine retinal vascular endothelium, which progressively decreased after the onset of diabetes. Furthermore, in an STZ-induced diabetic model, genetic loss of APN resulted in accelerated induction of early pathological features of DR, represented by increased vascular permeability, indicating the potential action of APN against DR progression.

## Results

### Localization of APN protein in the retinal vasculature

**Figure 1:**
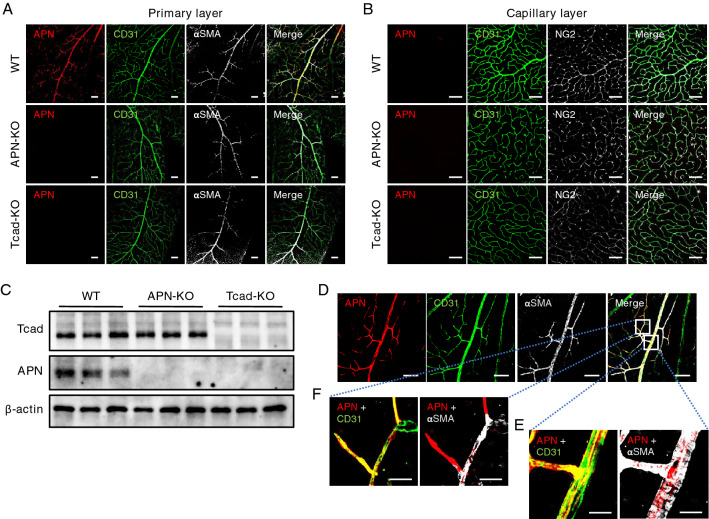
Localization of adiponectin in the mouse retina. (**A**, **B**) Representative images of retinal whole-mount immunofluorescence staining of male wild-type (WT), adiponectin knockout (APN-KO), and T-cadherin knockout (Tcad-KO) mice. (**A**) Triple immunostaining for APN (red), CD31 (green), and αSMA (gray) in the primary layer. Scale bar = 100 μm. (**B**) Triple immunostaining for APN (red), CD31 (green), and NG2 (gray) in the capillary layer. Scale bar = 100 μm. (**C**) Immunoblots for APN, Tcad, and β-actin proteins extracted from whole retinas of WT, APN-KO, and Tcad-KO mice. Original blots/gels are presented in Supple. Fig. 1. (**D**) Representative images of triple immunostaining for APN (red), CD31 (green), and αSMA (gray) of retinas from WT mice. Scale bar = 100 μm. (**E**, **F**) High magnification images of the arteriole (**E**) and the transitional zone between the arteriole and the capillary (**F**). Scale bar = 25 μm.

Firstly, we conducted immunofluorescence staining on retinal flat mounts to assess APN localization in the mouse retina. Figure 1A,B show representative immunofluorescence images for APN, alpha-smooth muscle actin (αSMA, a marker of vascular smooth muscle cells), and CD31 (a marker of vascular endothelial cells) in the primary layer and those for APN, neuron-glia antigen 2 (NG2, a marker of pericytes), and CD31 in the capillary layer, respectively. In wild-type (WT) mice, APN signal was mainly detected along retinal arterioles, but not venules, in the primary layer (Fig. 1A, upper panels), while it was hardly detectable in the capillary layer (Fig. 1B, upper panels). Such APN signal was completely diminished in T-cadherin knockout (Tcad-KO) mice (Fig. 1A,B, bottom panels) similar to APN knockout (APN-KO) mice (Fig. 1A,B, middle panels). Immunoblotting for T-cadherin and APN in the whole retinas is shown in Fig. 1C. Consistent with immunofluorescence results, both T-cadherin and APN proteins were detectable in whole retinal lysates of WT mice, whereas APN was barely detectable in Tcad-KO mice (Fig. 1C, unedited membranes are shown in Supple. Fig. 1). Under high magnification of retinal flat mount of WT mice, APN was found to be merged with CD31, rather than with αSMA, in the retinal arterioles of WT mice (Fig. 1D,E). We also observed APN signal, together with CD31, in the transitional zone between arterioles and capillaries in which αSMA was no longer detectable (Fig. 1D,F). These results indicate that APN protein was localized on the vascular endothelium of the murine retina in a T-cadherin-dependent manner.

### Decreased APN accumulation in the diabetic retinal vasculature

**Figure 2:**
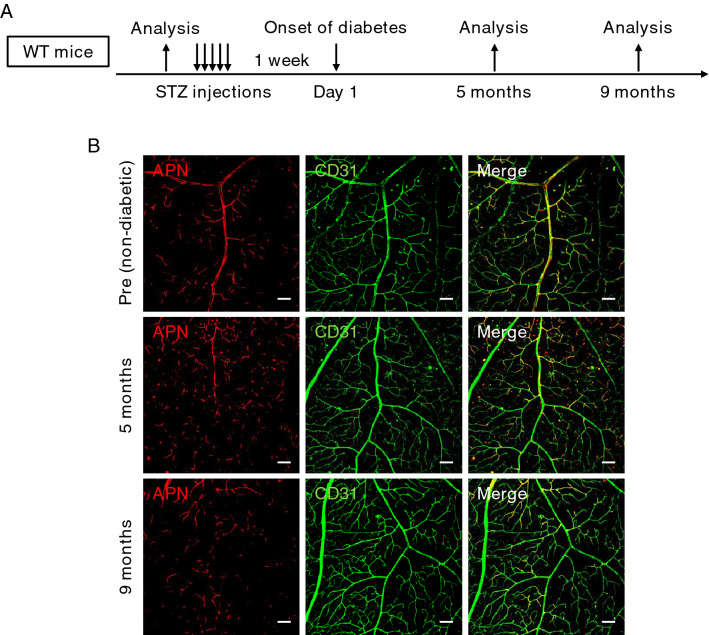
Changes of retinal adiponectin in streptozotocin-induced diabetic mice. (**A**) Streptozotocin (STZ) was intraperitoneally administered to 12-week-old male wild-type (WT) mice for 5 consecutive days. At 1 week after the last injection of STZ, mice with fasting blood glucose levels above 300 mg/dL were considered to be in a diabetic state and were selected for experimentation. Retinas were analyzed before STZ injection (Pre, non-diabetic) and 5 months and 9 months after the onset of diabetes. (**B**). Representative images of immunostaining for adiponectin (APN) (red) and CD31 (green) in the primary layer. Scale bar = 100 μm.

Next, an STZ-induced diabetic mouse model was used to examine quantitative change in APN protein accumulation in the retinal vasculature under diabetic conditions (Fig. 2A). As shown in Fig. 2B, immunofluorescence staining showed that APN in retinal arterioles was reduced at 5 months after the onset of diabetes, compared to non-diabetic mice. This retinal APN reduction had intensified by 9 months after the onset of diabetes, while no significant changes were observed in CD31 staining (Fig. 2B), indicating that APN accumulation in the retinal vascular endothelium progressively decreased during the course of diabetes.

### Effects of dapagliflozin on retinal APN and vascular permeability in STZ-induced diabetic mice

Increased retinal vascular permeability has been reported to be observed at the early stage of DR in mice and humans, which triggers the disease progression of DR^15,16^. We thus evaluated retinal vascular leakage in STZ-induced diabetic mice by intracardiac injection of Hoechst 33258 (molecular weight of 534 Da) and fluorescein isothiocyanate (FITC)-dextran (molecular weight of 3000–5000 Da). In this assay, extravascular Hoechst signals could be detected when circulating Hoechst leaked out of blood vessels and was incorporated into the nuclei of perivascular cells. Under the non-diabetic state (before STZ injections), FITC-dextran was distributed within vessels and Hoechst staining was observed only in the nuclei of endothelial cells (Supple. Fig. 2A). Either Hoechst or FITC-dextran was hardly detected in the extravascular space at 1 week after the onset of diabetes, whereas, by 6 weeks, extravascular Hoechst-positive, but not FITC-dextran-positive, regions were observed mainly around the bifurcations of peripheral arterioles (Supple. Fig. 2A). These results suggest that microvascular permeability for small molecules occurred at a relatively early stage after the onset of diabetes.

**Figure 3:**
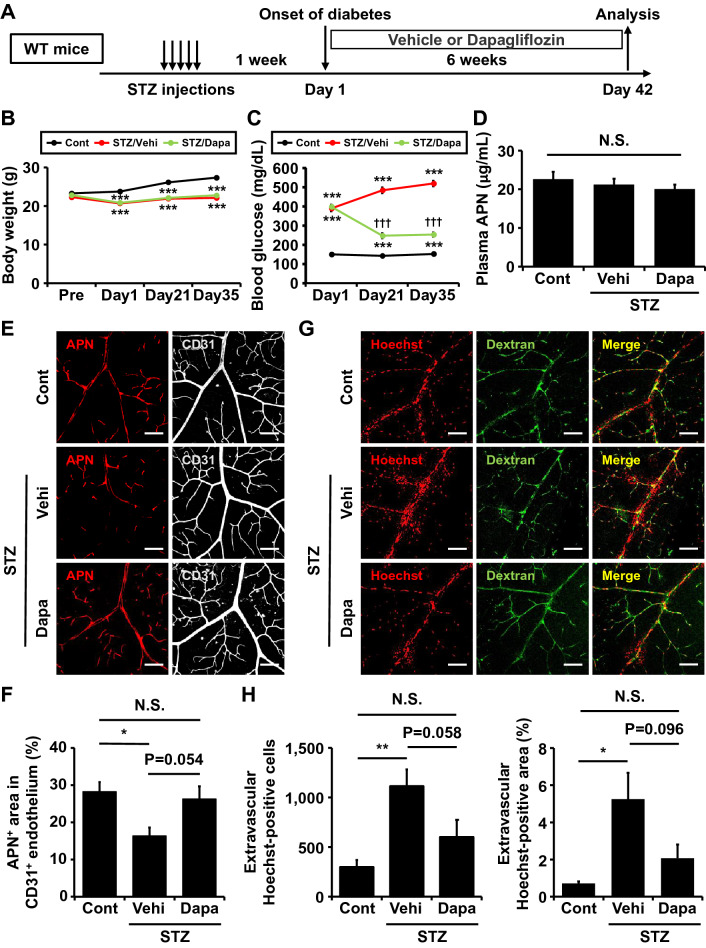
Glucose-lowering effects of dapagliflozin on retinal adiponectin and vascular permeability in streptozotocin-induced diabetic mice. (**A**) After the onset of streptozotocin (STZ)-induced diabetes (Day 1), 8-week-old male wild-type (WT) mice were treated with vehicle (STZ/Vehi) or dapagliflozin (STZ/Dapa) for 6 weeks. To evaluate retinal vascular permeability, a mixed solution of Hoechst and fluorescein isothiocyanate (FITC)-dextran was transcardially injected into each mouse, and retinas were removed at 5 min after injection. Mice without STZ injection were also analyzed as a control (Cont). (**B**, **C**) Changes in body weights (**B**) and blood glucose levels under 4-h fasting conditions (**C**). n = 14 for Cont, n = 19 for STZ/Vehi, and n = 18 for STZ/Dapa. Data are the mean ± SEM; ***P < 0.001 vs Cont and ^†††^P < 0.001 vs STZ/Vehi (one-way ANOVA with Tukey’s post hoc test). (**D**) Plasma adiponectin (APN) concentrations at day 35. n = 14 for Cont, n = 15 for STZ/Vehi, and n = 16 for STZ/Dapa. Data are the mean ± SEM; N.S., not significant (one-way ANOVA with Tukey’s post hoc test). (**E**) Representative images of retinal whole-mount immunofluorescence staining for APN (*red*) and CD31 (*gray*). Scale bar = 100 μm. (**F**) The % area of the APN-positive region in CD31-positive endothelium. Quantification was performed using two randomly obtained sections for each mouse. n = 6 for Cont, n = 7 for STZ/Vehi, and n = 8 for STZ/Dapa. Data are the mean ± SEM; *P < 0.05. N.S., not significant (one-way ANOVA with Tukey’s post hoc test). (**G**) Representative immunofluorescence images for Hoechst (red) and FITC-dextran (green). Scale bar = 100 μm. (**H**) Extravascular Hoechst-positive cell numbers (left) and % area (right) were calculated using two randomly obtained sections for each mouse. n = 8 for Cont, n = 12 for STZ/Vehi, and n = 10 for STZ/Dapa. Data are the mean ± SEM; *P < 0.05 and **P < 0.01. N.S., not significant (one-way ANOVA with Tukey’s post hoc test).

Next, we investigated blood glucose-lowering effects on retinal APN accumulation and vascular permeability in diabetic mice. After the induction of diabetes, STZ-injected mice were treated with vehicle (STZ/Vehi group) or dapagliflozin, a selective sodium-glucose co-transporter 2 (SGLT2) inhibitor, (STZ/Dapa group) for 42 days (Fig. 3A). Compared to non-diabetic control mice, a similar body weight reduction was observed in both the STZ/Vehi and STZ/Dapa groups (Fig. 3B), whereas blood glucose levels were significantly reduced to below 300 mg/dL by the administration of dapagliflozin (Fig. 3C, STZ/Vehi versus STZ/Dapa). Plasma APN levels did not differ among the three groups (Fig. 3D). Six weeks of hyperglycemia was associated with a significant reduction of APN on retinal arterioles compared to control mice (Fig. 3E,F, Cont versus STZ/Vehi), while APN accumulation was preserved in the retina of the STZ/Dapa group (Fig. 3E,F, STZ/Vehi versus STZ/Dapa). As shown in immunofluorescence images (Fig. 3G), extravascular Hoechst-positive regions were increased in STZ-induced diabetic mice (Cont versus STZ/Vehi), but were reduced by dapagliflozin treatment (STZ/Vehi versus STZ/Dapa). Treatment of diabetic mice with dapagliflozin tended to reduce extravascular Hoechst-positive cells (Fig. 3H, left) and area (Fig. 3H, right). Collectively, glucose-lowering treatment with dapagliflozin prevented a decrease in retinal APN accumulation and an increase in retinal vascular permeability.

**Figure 4:**
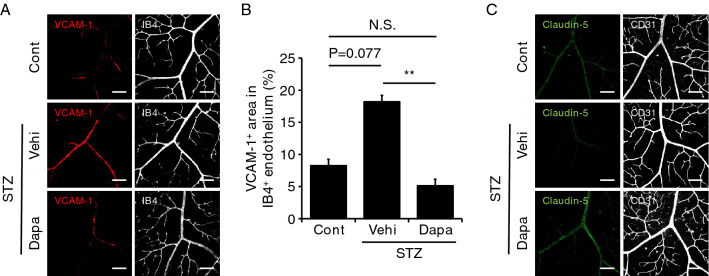
Glucose-lowering effects of dapagliflozin on vascular cellular adhesion molecule-1 and claudin-5 expression in the retinal vascular endothelium. After the onset of streptozotocin (STZ)-induced diabetes, 8-week-old male wild-type (WT) mice were treated with vehicle (STZ/Vehi) or dapagliflozin (STZ/Dapa) for 6 weeks. Mice without STZ injection were also analyzed as a control (Cont). Immunofluorescence staining was conducted using retinal whole-mount from each mouse. (**A**) Representative immunofluorescence images for vascular cellular adhesion molecule-1 (VCAM-1) (red) and isolectin B4 (IB4) (gray). Scale bar = 100 μm. (**B**) The % area of the VCAM-1-positive region in IB4-positive endothelium. Quantification was performed using two randomly obtained sections for each mouse. n = 4 for Cont, n = 7 for STZ/Vehi, and n = 6 for STZ/Dapa. Data are the mean ± SEM; **P < 0.01. N.S., not significant (one-way ANOVA with Tukey’s post hoc test). (**C**) Representative immunofluorescence images for claudin-5 (green) and CD31 (gray). Scale bar = 100 μm.

In both mice and humans, upregulation of endothelial cell adhesion molecules such as vascular cellular adhesion molecule-1 (VCAM-1) is recognized as one of the causes associating with DR development^17–19^. In the isolectin B4 (IB4)-labeled endothelium of retinal arterioles, VCAM-1 expression was tended to increase in diabetic mice compared to control mice (Fig. 4A,B, Cont versus STZ/Vehi), but was significantly suppressed by dapagliflozin (Fig. 4A,B, STZ/Vehi versus STZ/Dapa). In contrast to VCAM-1, expression of claudin-5, the major tight-junction molecule in endothelial cells of BRB^20^, was reduced in the STZ/Vehi group (versus Cont group), but was restored in the STZ/Dapa group (versus STZ/Vehi group) (Fig. 4C).

### Early progression of diabetic retinal vascular abnormalities in APN-deficient mice

**Figure 5:**
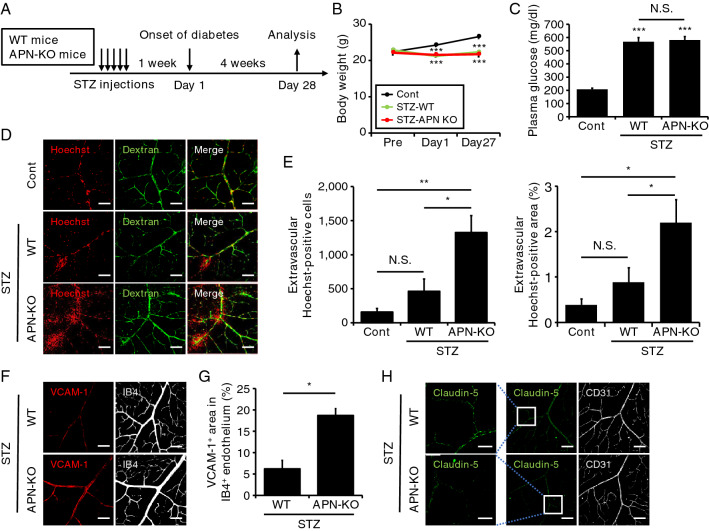
Impact of adiponectin deficiency on retinal vascular damage in streptozotocin-induced diabetes. (**A**) Streptozotocin (STZ) was intraperitoneally administered to 8-week-old male wild-type (WT) and adiponectin knockout (APN-KO) mice for 5 consecutive days. Mice were analyzed at 4 weeks after the onset of diabetes (Day 1). To evaluate retinal vascular permeability, a mixed solution of Hoechst and fluorescein isothiocyanate (FITC)-dextran was transcardially injected into each mouse, and retinas were removed at 5 min after injection. WT mice without STZ injection were also analyzed as control (Cont). (**B** and **C**) Changes in body weights (**B**) and plasma glucose concentrations under 4-h fasting conditions at Day 27 (the day before analysis). n = 6 for Cont, n = 9 for STZ-WT, and n = 8 for STZ-APN-KO. Data are the mean ± SEM; ***P < 0.001 vs Cont. N.S., not significant (one-way ANOVA with Tukey’s post hoc test). (**D**) Representative immunofluorescence images for Hoechst (red) and FITC-dextran (green). Scale bar = 100 μm. (**E**) Extravascular Hoechst-positive cell numbers (left) and % area (right) were calculated using two randomly obtained sections for each mouse. n = 6 for Cont, n = 9 for STZ-WT, and n = 8 for STZ-APN-KO. Data are the mean ± SEM; *P < 0.05 and **P < 0.01. N.S., not significant (one-way ANOVA with Tukey’s post hoc test). (**F**) Representative immunofluorescence images for vascular cellular adhesion molecule-1 (VCAM-1) (red) and isolectin B4 (IB4) (gray). Scale bar = 100 μm. (**G**) The % area of the VCAM-1-positive region in IB4-positive endothelium. Quantification was performed using two randomly obtained sections for each mouse. n = 4 for STZ-WT and n = 3 for STZ-APN-KO. Data are the mean ± SEM; *P < 0.05 (two-tailed Student’s t-test). (**H**). Representative immunofluorescence images for claudin-5 (green) and CD31 (gray). Scale bar = 25 μm (left panels) and 100 μm (middle and right panels).

Finally, since the APN reduction in the retinal endothelium accompanied early diabetic vascular changes (Figs. 3 and 4), we investigated the role of APN in the development of DR using APN-KO mice. Retinas from WT and APN-KO mice were immunohistochemically analyzed at 4 weeks after the onset of STZ-induced diabetes (Fig. 5A). Neither body weight nor plasma glucose levels differed between WT and APN-KO mice following STZ injection (Fig. 5B,C). Retinal vascular permeability in APN-KO mice was not altered under the non-diabetic state (Supple. Fig. 2B). However, compared to diabetic WT mice, diabetic APN-KO mice exhibited a significant increase in vascular permeability, determined by extravascular Hoechst-positive cells and area, after only 4 weeks of hyperglycemia (Fig. 5D,E). Additionally, hyperglycemia-induced upregulation of retinal VCAM-1 was significantly greater in retinas of APN-KO mice than in WT mice (Fig. 5F,G). Reflecting the increased vascular permeability, diabetic APN-KO mice showed a strong reduction in endothelial claudin-5 expression, notably in peripheral arterioles (Fig. 5H). These results indicate that APN deficiency was associated with a rapid progression of DR.

## Discussion

Our major findings in the present study were as follows: (1) in retina, APN existed mainly in the vascular endothelium of arterioles. Such APN localization was undetectable in Tcad-KO mice. (2) The amount of APN protein in the retinal vascular endothelium was significantly reduced in STZ-induced diabetic mice, accompanied by increased vascular permeability and reduced claudin-5 expression. (3) Retinal vasculature of APN-KO mice was more fragile than WT mice under hyperglycemic conditions.

Recent studies, including our own, have shown T-cadherin-dependent APN accumulation in various types of tissues and cells^8–11,21,22^. The present study clearly demonstrated that APN was predominantly localized in the vascular endothelium of retinal arterioles. Retinal whole-mount immunostaining for T-cadherin cannot be performed due to the lack of commercially available antibodies suitable for immunostaining, but retinal APN was absent in Tcad-KO mice (Fig. 1A,C). Thus, it can be concluded that APN exists in retinal arterioles by binding to T-cadherin. On the contrary, APN signal was hardly detectable in peripheral capillaries and venules. This result suggests a gradual decline of endothelial T-cadherin expression associated with the transition from arterioles to capillaries, which is compatible with recent single cell RNA-sequencing data from brain endothelial cells (BECs)^23^. We recently reported that, in kidney, both APN and T-cadherin were observed in platelet-derived growth factor receptor beta (PDGFRβ)-positive pericytes of peritubular capillaries, but not in glomerular or peritubular capillary endothelium^11^. However, in retina, no APN signal was found in NG2-positive pericytes in the capillary layer (Fig. 1B). These observations suggest that the expression patterns of T-cadherin and APN are not always similar in these perivascular cells.

The significant roles of APN in diabetic patients with DR are still controversial. Low plasma APN concentrations were shown to be associated with the severity of DR in patients with type 2 diabetes^24^. On the other hand, another observational study reported that both serum and aqueous humor APN levels were positively correlated with DR development and progression^25^. Since serum APN is increased with a decline in estimated glomerular filtration rate (eGFR)^26^, complications of advanced diabetic kidney disease (DKD) could account for this discrepancy. Interestingly, we found that retinal vascular APN was progressively reduced from the early stage of DR (6 weeks after the onset of STZ-induced diabetes) when plasma APN levels were still unchanged (Fig. 3D,E). This observation was thought to be hyperglycemia-dependent because glucose-lowering treatment with dapagliflozin prevented it (Fig. 3E). We previously demonstrated that T-cadherin deficiency resulted in significantly higher circulating levels of APN (4- to 5-fold compared with wild-type mice), probably due to the inability of APN to bind to T-cadherin-expressing tissues and cells^27^, and failed to benefit from protective effects of APN on cardiovascular diseases, including cardiac hypertrophy^9^ and atherosclerosis^8^. Our previous study also showed a significant decrease in T-cadherin protein in the aortas of genetically diabetic and obese *db/db* mice^28^. Thus, it is possible that T-cadherin expression in the retinal vascular endothelium is reduced under diabetic and/or hyperglycemic states. However, further investigation is required to determine whether quantitative changes in retinal vascular APN under diabetic conditions are also mediated via a T-cadherin-dependent mechanism.

The direct effects of SGLT2 inhibitors on DR progression are of particular interest in clinical settings because this class of antihyperglycemic agents has the potential to reduce the risk of DKD, partly independent of glycemic control^29^. Although we could not detect SGLT2 mRNA expression in whole retinas (data not shown), there are a few reports describing its expression in vascular endothelial cells^30^ and retinal pericytes^31^. It is also postulated that low-grade hyperketonemia observed during the treatment with SGLT2 inhibitors improves retinal hypoxia associated with DR^32^. Therefore, it cannot be ruled out that dapagliflozin exerted beneficial effects on altered vascular permeability and APN accumulation in diabetic retinas, beyond its glucose-lowering action.

To date, several clinical studies have suggested protective effects of APN against the development of DR. In the early phase of DR, circulating APN levels were positively correlated with retinal blood flow and negatively correlated with retinal arterial vascular resistance^33^. This clinical observation was supported by a previous in vitro study that APN elicited dilation of the retinal arterioles via the production of nitric oxide from the endothelium^34^. Experimentally, APN-KO mice displayed severe pathological retinal neovascularization in a neonatal ischemia-induced retinopathy model, which was attenuated by adenovirus-mediated overexpression of APN through modulation of inflammatory responses and leukocyte adhesion to the retinal endothelium^35^. However, to our knowledge, the present study is the first report providing a direct link between APN and diabetic retinal vascular abnormalities. Interestingly, extravascular Hoechst leakage in STZ-induced diabetic mice was largely observed around branches of peripheral arterioles where APN accumulation was reduced (Fig. 3E,G). Under non-diabetic conditions, no alteration of retinal vasopermeability was observed in APN-KO mice, which is consistent with previous results demonstrating that APN deficiency did not influence physiological characteristics, including insulin resistance^1^, cardiac function^2^, and renal vascular permeability^11^. On the contrary, APN-KO mice exhibited severely increased vascular permeability under a relatively short term of hyperglycemia, despite similar blood glucose levels to diabetic WT mice. This observation may partly be explained by a significant increase in endothelial VCAM-1 expression in the retinas of diabetic APN-KO mice because leukocyte activation through its endothelial counterparts, VCAM-1 and intercellular adhesion molecule-1 (ICAM-1), is considered to be an important contributory factor towards inflammation and BRB leakage in the diabetic retinal microvasculature^36^. Regarding the interaction of APN with cell adhesion molecules, Ouchi et al. previously showed that APN inhibited tumor necrosis factor-alpha (TNF-α)-induced upregulation of VCAM-1 and ICAM-1 mRNA by suppressing nuclear factor kappaB (NF-kappaB) signaling in human aortic endothelial cells^37^. As such, it could be inferred that APN prevented VCAM-1 mediated retinal leukostasis and the subsequent breakdown of the BRB. In addition, intracellular accumulation of ceramide, a potential intracellular lipid mediator, is thought to be involved in the pathogenesis of diabetic microvascular complications through the process of lipotoxicity, including inflammation, oxidative stress, and cell death^38^. Importantly, we demonstrated the T-cadherin-mediated effect of APN on promoting exosome biogenesis and secretion, through which APN exerted ceramide efflux activity, followed by reducing cellular ceramide contents in cultured endothelial cells^39^. APN may exhibit its protective actions against diabetes-induced retinal vascular damage by enhancing exosome production.

Claudin-5 is a critical component of the BRB that controls vasopermeability in association with other tight-junction proteins, such as occludin and zonula occludens-1 (ZO-1)^20^. Several studies have demonstrated that claudin-5 redistribution occurs in the diabetic retina^40–42^. We observed extravascular leakage of Hoechst, but not FITC-dextran, together with a reduction in endothelial claudin-5 in retinas of diabetic mice, indicating a causal relationship between altered claudin-5 expression and increased vascular permeability. This result is in line with previous findings that claudin-5-deficient mice showed size-selective molecular loosening of the blood–brain barrier (BBB), allowing the passage of small molecules (< 800 Da)^43^. Under pathological conditions, post-translational regulation of claudin-5 has been suggested to be involved in its reduction from the surface of endothelial cells^44^. Metalloproteinase-2 and -9 (MMP-2/9), whose activities are known to be increased in the plasma of type 1 diabetic patients^45,46^, have the potential for claudin-5 degradation^47,48^, while caveolae-mediated internalization of claudin-5 can be promoted by the pro-inflammatory cytokine CCL-2^49^. Circulating APN levels were negatively correlated with plasma MMP-2 and MMP-9 activities in patients with obesity^50^ and coronary artery disease (CAD)^51^, and APN inhibited angiotensin-II-induced cardiac fibrosis through the suppression of MMP-2/9 activities in mice^52^ Therefore, the absence of APN-dependent anti-inflammatory and anti-MMPs effects might accelerate retinal claudin-5 degradation and the significant increase in vascular permeability, both of which were observed in STZ-induced diabetic APN-KO mice. In mouse models of retinal vein occlusion and oxygen-induced retinopathy, Nishinaka et al. recently reported that APN expression was increased at the occluded site of the veins in damaged retina, and an intravitreal injection of anti-APN antibody improved retinal edema and ischemia^53^. However, at least in our model of STZ-induced diabetes, we could not detect APN mRNA expression in retina (data not shown) and retinal APN protein was significantly decreased (Figs. 2B and 3E). Although the reasons for this discrepancy are uncertain, these conflicting results of APN’s effects on retinal vascular diseases might be explained by the differences in the models used in the experiments.

In conclusion, the present study, for the first time, showed a significant localization of APN in the retinal vasculature and its possible role in preventing vascular damage caused by hyperglycemia. Hyperglycemia-induced reduction of retinal APN might be associated with the development of DR.

## Methods

### Animals

Male C57BL/6J WT mice were purchased from CLEA Japan (Tokyo, Japan). APN-KO and Tcad-KO mice were generated as previously described^1,54^ and were bred on a C57BL/6J background. Mice were housed in 22 ℃ in 12-h light/12-h dark cycle (lights on from 8:00 a.m. to 8:00 p.m.).

For the diabetic mouse model, STZ (Wako Pure Chemical, Osaka, Japan), dissolved in 0.05 M citrate buffer (pH 4.5), was intraperitoneally injected into 8- or 12-week-old male mice under 4-h fasting conditions at doses of 60 mg/kg for 5 consecutive days. One week after the last injection of STZ, blood glucose was measured with a glucose analyzer under 4-h fasting conditions. Mice with blood glucose levels above 300 mg/dL were considered to be in a diabetic state and selected for experimentation. The administration of dapagliflozin (Sigma-Aldrich, St Louis, MO) to diabetic mice was conducted as previously described^55^. Briefly, stock solution of dapagliflozin (125 mg/mL in 100% ethanol) was diluted to a final concentration of 0.02 mg/mL in distilled water. Then, dapagliflozin solution was given in the drinking water, commencing from the day when hyperglycemia was confirmed. Water drinking bottles were changed twice a week.

In all experiments, mice were anesthetized by intraperitoneal injection of a mixture of medetomidine (0.3 mg/kg), midazolam (4 mg/kg), and butorphanol (5 mg/kg) before tissue dissection. The experimental protocol was approved by the Ethics Review Committee for Animal Experimentation of Osaka University, Graduated School of Medicine, and carried out in accordance with the Institutional Animal Care and Use Committee Guidelines of Osaka University. This study also conformed to the Guide for the Care and Use of Laboratory Animals published by the US National Institutes of Health and the ARRIVE guidelines.

### Immunofluorescence staining of mouse retinal flat mounts

Immunofluorescence staining of whole-mount retinas was performed as previously described^56^. In brief, eyes were excised from mice after transcardiac perfusion with ice-cold saline to eliminate the contamination of circulating APN. Isolated retina cups were fixed in 4% paraformaldehyde for 90 min at room temperature. After fixation, retinas were washed 3 times in phosphate-buffered saline (PBS) for 30 min at 4 ℃ and blocked with blocking buffer (0.5% Blocking Reagent (Roche, Basel, Switzerland) and 0.2% Triton X-100 in 10 mM Tris–HCl (pH 7.5)) for 1 h at room temperature. Then, the retina cups were incubated overnight at 4 ℃ with the following primary antibodies, diluted with 1% bovine serum albumin (BSA) and 0.1% Triton X-100 in PBS: goat anti-APN (1:100; R&D Systems, Minneapolis, MN), rabbit anti-αSMA (1:100; Abcam, Cambridge, MA), rabbit anti-NG2 (1:100; EMD Millipore, Billerica, MA), rat anti-CD31 (1:100; Invitrogen, Carlsbad, CA), rat anti-VCAM-1 (1:100; Santa Cruz Biotechnology, Santa Cruz, CA), Alexa Fluor 488 conjugated mouse anti-claudin-5 (1:100; Invitrogen), and Alexa Fluor 647 conjugated IB4 (1:100, Invitrogen). Chicken anti-Rabbit IgG conjugated Alexa 488 (Life Technologies, Gaithersburg, MD), Donkey anti-goat IgG conjugated Alexa 594 (Life Technologies), and chicken anti-rat IgG conjugated Alexa 647 (Life Technologies) were used as secondary antibodies. Subsequently, retina cups were flat-mounted for fluorescent microscopy. Images were acquired using an Olympus FV3000 confocal laser scanning microscope system (Olympus, Lake Success, NY). All images were obtained in the area 800–1800 μm from the optic disk.

For the quantitative assessment of VCAM-1- and APN expression in retinal vessels, two obtained squares (600 μm × 600 μm) for VCAM-1/IB4- and APN/CD31-staining sections were analyzed for each mouse. We calculated the % area of VCAM-1-positive or APN-positive regions to IB4-positive or CD31-positive regions (total vessel area), respectively, using ImageJ software. The same color tone in all images was measured at the same threshold.

### Assessment of retinal vascular permeability

Retinal vascular permeability was quantitatively evaluated by extravasation of Hoechst 33258 (Abcam), as previously described^33^. Briefly, PBS containing 200 μg/mL of Hoechst and 5 mg/mL of FITC-dextran (Sigma) (total volume 500 μL) was injected into the left ventricle of each mouse under anesthesia. Five min after injection, retina cups were enucleated and fixed in 4% paraformaldehyde for 90 min at room temperature. Then, retinal flat mounts were subjected to microscopy analysis using an Olympus FV3000 confocal laser scanning microscope system. All images were obtained in the area 800–1800 μm from the optic disk. Except for Hoechst signals incorporated into the nuclei of vascular endothelial cells, extravascular Hoechst-positive cells and areas were calculated using ImageJ software as follows: (1) to obtain the total vessel area, FITC-dextran distributed within blood vessels was extracted by removing the autofluorescence background (representative images were shown in Supple. Fig. 3A). (2) The total Hoechst-positive region was merged with the FITC-dextran-positive total vessel region (representative images are shown in Supple. Fig. 3B). (3) The FITC-dextran-positive total vessel image was subtracted from the combined image of the Hoechst and FITC-dextran-positive region (representative images are shown in Supple. Fig. 3C). (4) The obtained image of the extravascular Hoechst-positive region was analyzed by ‘Count’ and ‘% Area' of ImageJ’s Analyze Particles function. We analyzed two captured squares (600 μm × 600 μm) for each mouse.

### Immunoblotting

Western blot analysis was conducted as previously described^11^. Briefly, frozen retinas were lysed in radioimmunoprecipitation assay (RIPA) buffer containing protease inhibitor. Protein lysates, obtained after centrifugation at 15,000×*g* at 4 °C for 30 min, were boiled with sample buffer (2% SDS, 50 mM Tris–HCl, 10% glycerol, and 6.6% 2-mercaptoethanol) at 98 °C for 5 min. The same amount of protein samples was subjected to 4–20% gradient SDS-PAGE (Bio-Rad, Hercules, CA). The following primary antibodies were used: goat anti-APN (1:1000, R&D), goat anti-T-cadherin (1:1000, R&D), and rabbit anti-β-actin (Cell Signaling Technology, Beverly, MA). Chemiluminescence signals were visualized by ChemiDoc Touch TM and quantitated using Image Lab software (Bio-Rad).

### Measurement of blood parameters

Blood samples were collected from tail veins under 4-h fasting conditions. Plasma glucose levels were measured by Glucose CII-Test (Wako Pure Chemical). Plasma APN was measured using the mouse adiponectin enzyme-linked immunosorbent assay (ELISA) kit (Otsuka Pharmaceuticals, Tokyo, Japan) according to the manufacturer's protocol^8^.

### Statistics

Data were expressed as the means ± SEM. Differences between the groups were analyzed by Student’s unpaired t-test or one-way ANOVA followed by Tukey’s honestly significant difference (HSD) test for multiple comparisons. Values of P < 0.05 (two-tailed) were considered significant. All statistical analyses were performed using JMP Pro software 14.3 (SAS Institute, Cary, NC).

## Supplementary Information

Supplementary Legends.

Supplementary Figure 1.

Supplementary Figure 2.

Supplementary Figure 3.

## Data Availability

The data sets generated during this study are available from the corresponding author upon reasonable request.
