# Supplementary material for: Adiponectin accumulation in the retinal vascular endothelium and its possible role in preventing early diabetic microvascular damage
Source: Sci Rep. 2022 Mar 9;12:4159. doi: 10.1038/s41598-022-08041-2 (PMC8907357; doi:10.1038/s41598-022-08041-2)
Supplement: Supplementary file 1 — Supplementary Legends. [file 41598_2022_8041_MOESM1_ESM.docx]

**Supplementary Figure Legends**

**Supplementary Figure 1.** **Full unedited blots for Fig. 1C**

Each membrane was cut prior to hybridization with each primary antibody. Images in Fig. 1C were indicated by red rectangles.

**Supplementary Figure 2. Altered retinal vascular permeability caused by streptozotocin-induced diabetes.**

To evaluate retinal vascular permeability, a mixed solution of Hoechst and fluorescein isothiocyanate (FITC)-dextran was transcardially injected into each mouse. Then, retinas were removed 5 min later. (A and B) Representative images of retinal whole-mount immunofluorescence staining for Hoechst (*red*) and FITC-dextran (*green*). (A) Retinas from wild-type (WT) mice before STZ injection (Pre, non-diabetic) and 1 week and 6 weeks after the onset of streptozotocin (STZ)-induced diabetes. (B) Retinas from non-diabetic adiponectin knockout (APN-KO) mice. Scale bar = 100 μm.

**Supplementary Figure 3.** **Quantification of extravascular Hoechst-positive cell numbers and area.**

(A) Representative images of the fluorescein isothiocyanate (FITC)-dextran-positive total vessel region after removing the autofluorescence background using ImageJ software. (B) Representative combined images of the FITC-dextran-positive total vessel region and Hoechst-positive region. (C) Extravascular Hoechst-positive cell numbers and area were calculated by subtracting the FITC^+^ total vessel region from the FITC^+^/ Hoechst^+^ combined region. Scale bar = 100 μm.
