# Supplementary figures and images for: Adiponectin accumulation in the retinal vascular endothelium and its possible role in preventing early diabetic microvascular damage
Source: Sci Rep. 2022 Mar 9;12:4159. doi: 10.1038/s41598-022-08041-2 (PMC8907357; doi:10.1038/s41598-022-08041-2)

## Slide 1
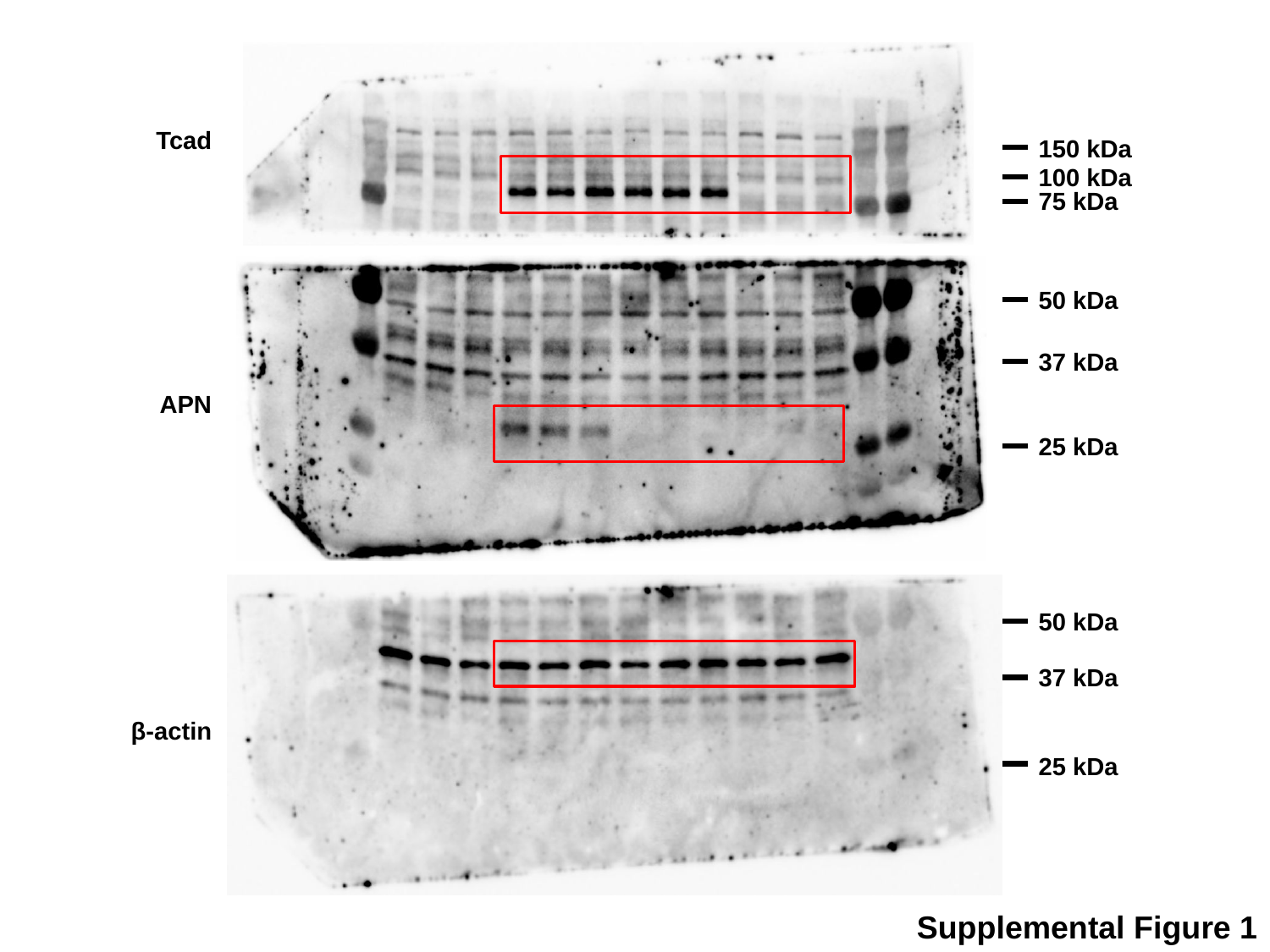

Tcad
150 kDa
100 kDa
75 kDa
50 kDa
37 kDa
APN
25 kDa
50 kDa
37 kDa
β-actin
25 kDa
Supplemental Figure 1

Supplement: Supplementary file 2 — Supplementary Figure 1. [file 41598_2022_8041_MOESM2_ESM.pptx]
