# Supplementary material for: Adiponectin accumulation in the retinal vascular endothelium and its possible role in preventing early diabetic microvascular damage
Source: Sci Rep. 2022 Mar 9;12:4159. doi: 10.1038/s41598-022-08041-2 (PMC8907357; doi:10.1038/s41598-022-08041-2)
Supplement: Supplementary file 3 — Supplementary Figure 2. [file 41598_2022_8041_MOESM3_ESM.pptx]

## Slide 1
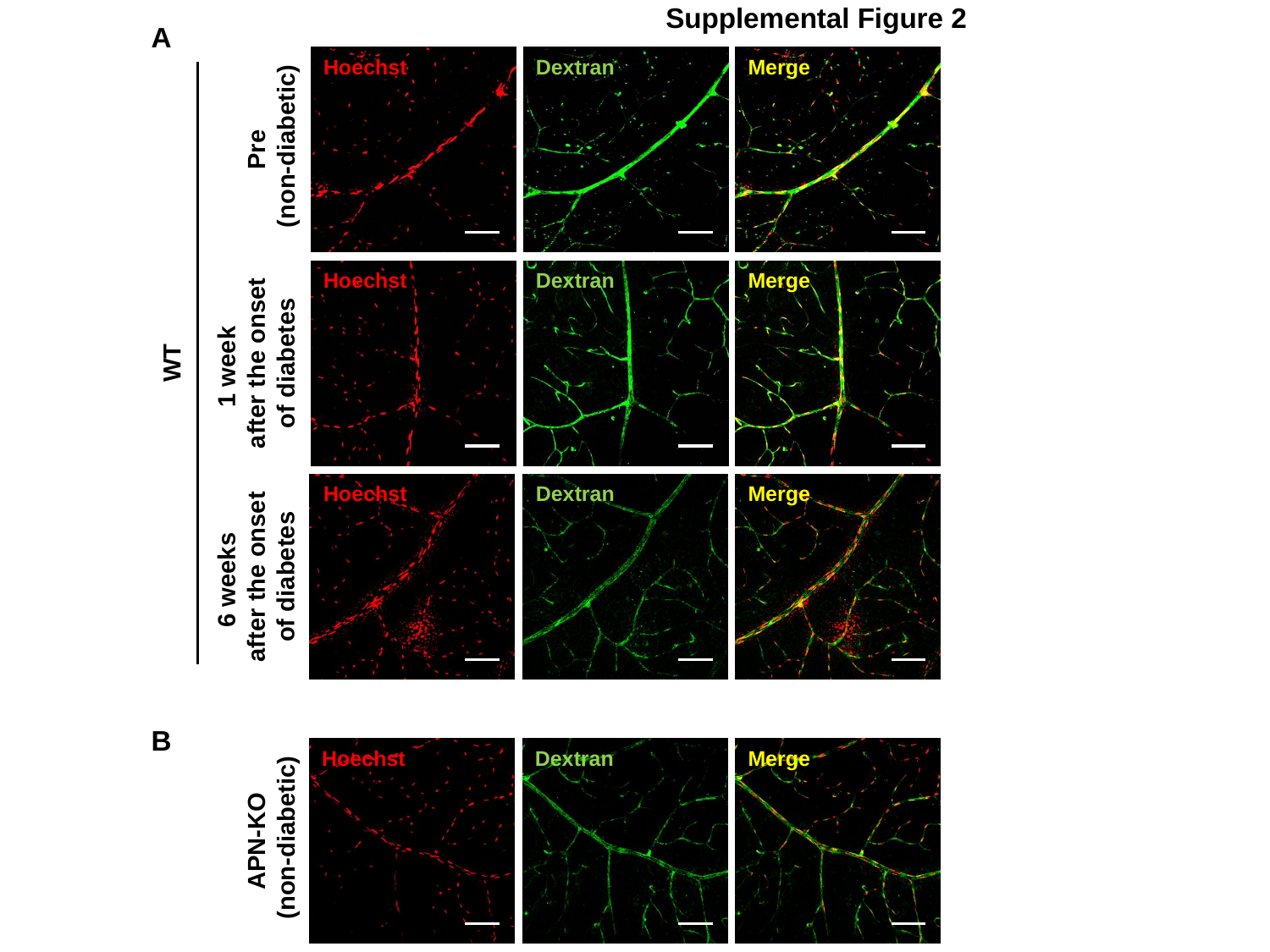

Supplemental Figure 2
A
Hoechst
Dextran
Merge
Pre
 (non-diabetic)
Hoechst
Dextran
Merge
1 week
after the onset
of diabetes
WT
Hoechst
Dextran
Merge
6 weeks
after the onset
of diabetes
B
Hoechst
Dextran
Merge
APN-KO
 (non-diabetic)
