# Supplementary material for: Adiponectin accumulation in the retinal vascular endothelium and its possible role in preventing early diabetic microvascular damage
Source: Sci Rep. 2022 Mar 9;12:4159. doi: 10.1038/s41598-022-08041-2 (PMC8907357; doi:10.1038/s41598-022-08041-2)
Supplement: Supplementary file 4 — Supplementary Figure 3. [file 41598_2022_8041_MOESM4_ESM.pptx]

## Slide 1
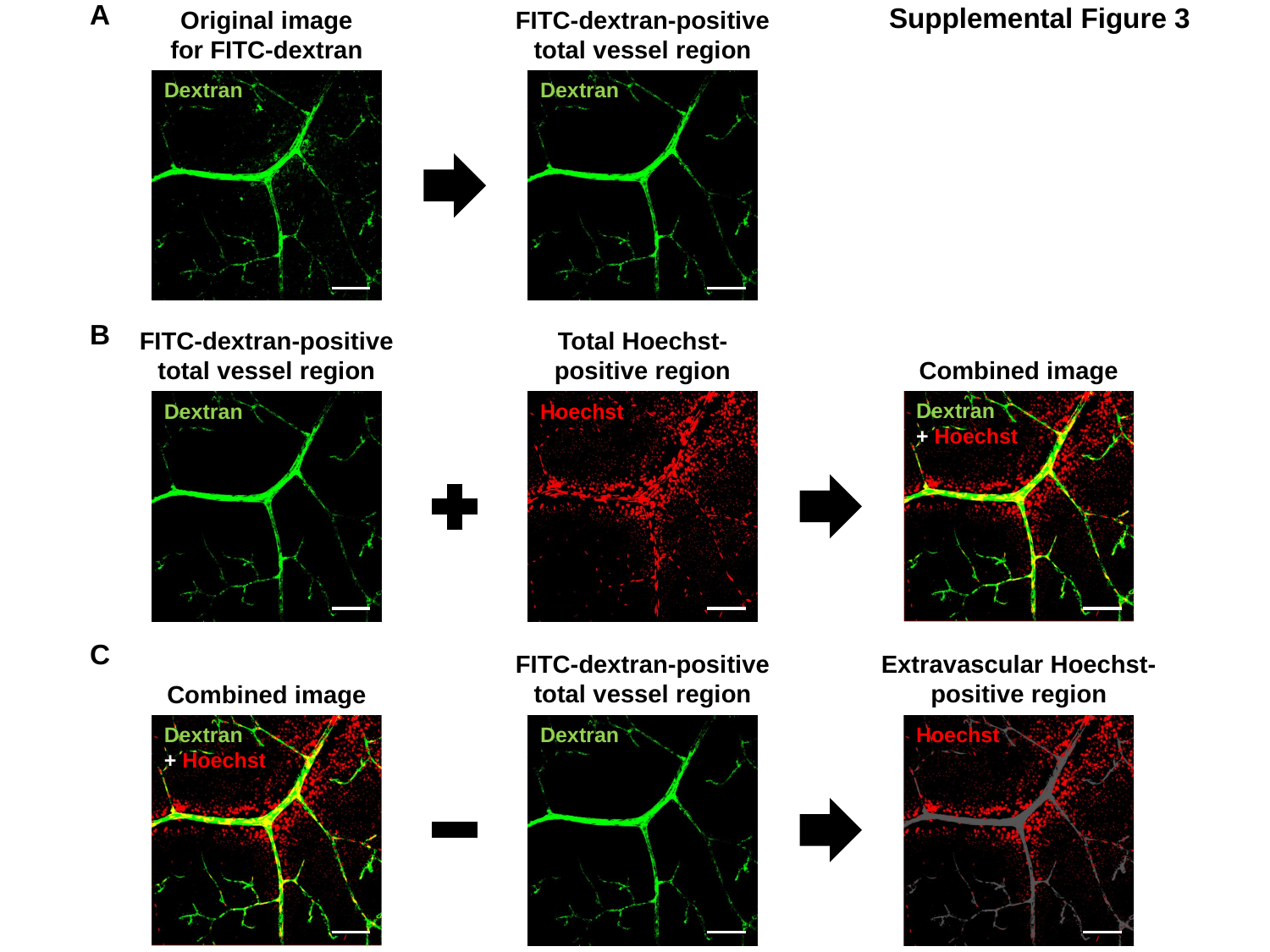

Supplemental Figure 3
A
Original image for FITC-dextran
FITC-dextran-positive total vessel region
Dextran
Dextran
B
FITC-dextran-positive total vessel region
Total Hoechst-positive region
Combined image
Dextran
Hoechst
Dextran
+ Hoechst
C
FITC-dextran-positive total vessel region
Extravascular Hoechst-positive region
Combined image
Dextran
+ Hoechst
Dextran
Hoechst
